# Effect of Steam Injection during Carbonation on the Multicyclic Performance of Limestone (CaCO_3_) under Different Calcium Looping Conditions: A Comparative Study

**DOI:** 10.1021/acssuschemeng.1c06314

**Published:** 2022-01-06

**Authors:** Juan Jesús Arcenegui Troya, Virginia Moreno, Pedro E. Sanchez-Jiménez, Antonio Perejón, José Manuel Valverde, Luis A. Pérez-Maqueda

**Affiliations:** † Instituto de Ciencia de Materiales de Sevilla, C. S. I. C.-Universidad de Sevilla, C. Américo Vespucio no 49, 41092 Sevilla, Spain; ‡ Departamento de Química Inorgánica, Facultad de Química, Universidad de Sevilla, Sevilla 41012, Spain; § Departamento de Electrónica y Electromagnetismo, Facultad de Física, Universidad de Sevilla, Avenida Reina Mercedes s/n, Sevilla, 41012 Spain

**Keywords:** Concentrated solar power, CO_2_ capture, Thermochemical energy storage, Calcium looping, Steam

## Abstract

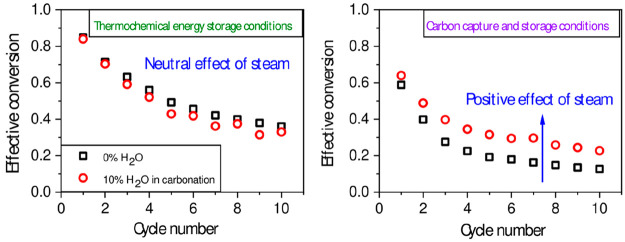

This study explores the effect of steam addition during carbonation on the multicyclic performance of limestone under calcium looping conditions compatible with (i) CO_2_ capture from postcombustion gases (CCS) and with (ii) thermochemical energy storage (TCES). Steam injection has been proposed to improve the CO_2_ uptake capacity of CaO-based sorbents when the calcination and carbonation loops are carried out in CCS conditions: at moderate carbonation temperatures (∼650 °C) under low CO_2_ concentration (typically ∼15% at atmospheric pressure). However, the recent proposal of calcium-looping as a TCES system for integration into concentrated solar power (CSP) plants has aroused interest in higher carbonation temperatures (∼800–850 °C) in pure CO_2_. Here, we show that steam benefits the multicyclic behavior in the milder conditions required for CCS. However, at the more aggressive conditions required in TCES, steam essentially has a neutral net effect as the CO_2_ uptake promoted by the reduced CO_2_ partial pressure but also is offset by the substantial steam-promoted mineralization in the high temperature range. Finally, we also demonstrate that the carbonation rate depends exclusively on the partial pressure of CO_2_, regardless of the diluting gas employed.

## Abstract 2

Steam injection can be used to enhance the calcium-looping performance in carbon capture and thermochemical energy storage applications.

## 1 Introduction

Climate change has evinced the need to mitigate CO_2_ emissions from fossil fuel combustion in power plants. The development of efficient CO_2_ capture technologies and the global deployment of renewable energy are keys to overcoming this challenge. The progress of renewable energies goes parallel with the development of efficient energy storage systems to avoid issues related to the intrinsic intermittency of natural energy sources, such as solar. With regards to this latter energy source, several systems have been proposed, including the use of molten salts and liquid metal oxides.^1,2^

In recent years, the calcium looping (CaL) process has aroused a great deal of interest as a potential avenue for reducing carbon emissions from postcombustion gases.^3^ The CaL-based technology relies on the reversible carbonation/calcination reaction of CaO/CaCO_3_:^4^
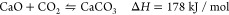
1

In the scheme proposed for carbon capture and storage (CCS), carbonation of CaO is conducted at around 650 °C, with a low CO_2_ concentration (∼15% vol as typical in postcombustion gases). To complete the cycle, after carbonation, CaCO_3_ particles are driven to the calciner reactor, where calcination is carried out at around 950 °C in a CO_2_ rich atmosphere (typically 70% vol).^5^ Then, CO_2_ released in calcination is stored, whereas the regenerated CaO is used in a new cycle.

Recently, the CaL-based technology has also been proposed for the thermochemical energy storage of concentrated solar power (CSP).^6^ The CaL-CSP technology presents certain advantages as compared to current commercial storage technologies based on sensible heat storage in molten salts. These include long-term energy storage and high energy densities (∼3.2 GJ/m^3^).^7^ Besides, natural CaO precursors such as limestone or dolomite are abundant, nontoxic, and cheap, which would facilitate the commercial deployment of the CaL-CSP technology.^8−12^

In the general scheme proposed for CaL-CSP, solar radiation is used to drive the endothermic decomposition of CaCO_3_ (calcination), as originally proposed by Flamant et al.^13^ The products of the reaction, CaO and CO_2_, are stored separately. When the power supply is required, they are brought back together to drive the exothermic reverse reaction (carbonation) that provides the heat needed for power production under demand. Afterward, the regenerated CaCO_3_ is again ready for a new calcination cycle.

In the CaL-CSP integration, heat to power efficiency is maximized by carrying out carbonation at high temperatures (around 850 °C), which may be quickly achieved in a pure CO_2_ atmosphere.^14^ High carbonation temperatures in CO_2_ ensure not only fast carbonation in short residence times but also allow for higher exergy efficiencies.^7,15^

A main caveat of the CaL-based technologies is the progressive loss of the CaO carbonation reactivity, essentially due to intense sintering at high temperatures and further promoted in a CO_2_-rich atmosphere.^16,17^ In order to preserve activity and mitigate CaO and CaCO_3_ sintering, mild calcination conditions are preferred, at around 750 °C in an inert gas.^14,18,19^ A further limiting mechanism evidenced mainly for particle sizes above ∼50 μm is pore plugging; carbonation at high temperatures under high CO_2_ partial pressure leads to the rapid formation of a thick CaCO_3_ layer on the CaO surface which blocks the pores and impedes CO_2_ from reaching the CaO unreacted core.^20−22^ Significant CaO deactivation eventually requires the removal of the spent sorbent to be replaced by fresh CaO precursor, thereby decreasing the efficiency and increasing the cost of these technologies.^23^ Therefore, much effort has been devoted to developing strategies for preventing the loss in CaO reactivity. Most methods rely on thermal and chemical pretreatments^24−28^ or even CaO modification using refractory additives.^29−31^

The influence of high-temperature steam on CaO reactivity has been amply studied in conditions compatible with CaL-CCS,^32−41^ as the combustion flue gases contain steam in 5–10% volume content for coal combustion, and up to 20% for oxy-fuel combustion.^33,34,36^ As a summary, Table 1 contains a selection of works exploring the influence of steam on the capture performance of CaO-based sorbents.

**Table 1:** Selected Literature on the Influence of Steam on the Capture Performance of CaO-Based Sorbents

authors (ref)	temperature (°C)	atmosphere	conclusions
Coppola et al.^32^	Car: 650	Car: 15% CO_2_, 10% steam, balanced by air	steam enhances sorbent reactivity
	Cal: 940	Cal: 70% CO_2_ balanced by air	
Donat et al.^33^	Car: 650	Car: 15% CO_2_ balanced by N_2_	steam enhances sorbent reactivity
	Cal: 900	Cal: 100% N_2_	
		0–20% steam	
Champagne et al.^34^	Car: 620	Car: 15% CO_2_ balanced by N_2_	steam enhances sorbent reactivity
	Cal: either 875 or 925	Cal: 60% CO_2_ balanced by N_2_	
		0–40% steam.	
Homsy et al.^35^	Car: 650	Car: 12% CO_2_ and 10% steam, balanced by N_2_	Steam negatively influences the capture performance of marble-derived CaO
	Cal: between 850 and 900	Cal: 30% CO_2_ and 13% steam, balanced by N_2_	
Manovic and Anthony^36^	Car: From 350 to 800	Car: 20% CO_2_ and 0–20% steam, balanced by N_2_	steam enhances sorbent reactivity
	Cal: 800 or 950, depending on the atmosphere	Cal: 100% N_2_ or 100% CO_2_	
Li et al.^37^	Car: From 400 to 700	Car: 15% CO_2_ and 2–20% steam, balanced by N_2_	steam enhances sorbent reactivity
	Cal: 850	Cal: 100% N_2_	
Kavosh et al.^38^	Car: 650	Car: 15% CO_2,_ 4% O_2_ and 6–20% steam, balanced by N_2_	steam enhances sorbent reactivity
	Cal: 950	Cal: 2% N_2_, 3% O_2_ and 28–78% steam, balanced by CO_2_	
Li et al.^43^	Car: 650	Car: 33% steam balanced by CO_2_	steam enhances sorbent reactivity
	Cal: 950	Cal: 20,40 and 60% balanced by CO_2_	
Arcenegui et al.^52^	Car: 850	Car: 100% CO_2_	steam enhances sorbent reactivity
	Cal: 680, 700 and 730	Cal: 0, 3% or 29% steam balanced by N_2_	
Lindén et al.^53^	Car: 400–550	Car:15% CO_2_, 0%, 3%, 10%, and 30%, balanced by N_2_	steam enhances sorbent reactivity
	Cal: 800	Cal: 100% N_2_	
Dong et al.^54^	Car: 650	Car:20% CO_2_, 0%,10%,20% and 40%, balanced by N_2_	steam enhances sorbent reactivity
	Cal: 900	Cal: 100% N_2_	

It has been demonstrated that the presence of steam accelerates CaCO_3_ calcination. Some authors attribute this effect to an increase in the heat transfer coefficient due to the higher thermal conductivity of steam as compared to N_2_.^42−44^ Other authors have reported a catalytic effect of steam.^45−49^ Giammaria and Lefferts observed a decrease in the apparent activation energy and attributed the acceleratory effect of steam to the formation of hydrogen carbonate ions as intermediates.^50^ Alternatively, faster calcination has been ascribed to steam-induced changes in crystal growth and surface reactivity.^51^ In any case, it has been proposed the acceleratory effect of steam injection can be used to lower the temperature needed to fully calcine the CaCO_3_ in the short residence time required in practice, which would lead to important energy savings. Moreover, attaining full calcination at lower temperatures would alleviate any sintering-induced deactivation thus improving multicyclic performance.^39,42−44,52^

An open question remains on the overall impact of steam on the multicyclic performance of CaO-based sorbents. It is widely acknowledged that steam substantially promotes sintering,^16,55^ but the implication of this effect on CaO carbonation is still controversial. Thus, while several authors have reported that steam addition offers negligible or even negative influence on CO_2_ carrying capacity,^56,57^ other researchers have otherwise observed a beneficial effect.^33,34,36,37,53,54,58^ Furthermore, Donat et al. observed that the addition of steam enhances multicyclic activity regardless of whether steam is injected during calcination, carbonation, or in both stages.^33^ In the latter cases, the improvement is attributed to the synergistic action of different mechanisms.^34^ Thus, the enhanced particle sintering during calcination in the presence of steam gives rise to a CaO structure with large pores (∼1 μm), which are less susceptible to pore plugging, thereby favoring the subsequent carbonation stage.^39,52^ On the other hand, as Manovic and Arias observed, steam has no influence on the carbonation rate during the initial fast reaction-controlled regime, but it strongly promotes the rate of conversion during the subsequent slow diffusion-controlled regime.^36,59^ Thus, the benefit of steam on CaO reactivity is explained mainly by an enhancement of the solid-state diffusion through the carbonate layer.^33,36,60,61^ Li et al. pointed out that enhanced carbonation might be attributed to the formation of OH^–^ ions after H_2_O dissociation.^37^ As a consequence of the enhanced solid-state diffusion, the microstructure of CaCO_3_ formed during carbonation in the presence of steam lacks nanosized porosity.^36^ Nonetheless, Homsy et al. has recently pointed out that this effect is by no means universal to all CaO-based sorbents but would ultimately depend on the calcium precursor’s microstructure.^35^

On the other hand, it is generally agreed that CaO hydration to obtain Ca(OH)_2_ can be used to enhance the extension of carbonation. As Ca(OH)_2_ is more reactive toward CO_2_ than CaO, some authors have suggested the transient formation of Ca(OH)_2_ as the reason behind the improvement of conversion.^57,62^ However, while reasonable at intermediate temperatures, it is debatable that such mechanism remains at temperatures over 500 °C, when the formation of Ca(OH)_2_ is no longer thermodynamically favored. In such cases, the enhanced carbonation should be attributed to steam-enhanced solid-state diffusion in the presence of steam.^33,36^

As it can be observed in Table 1, to this date, most experiments with steam were carried out in conditions relevant for CCS, in environments with low CO_2_ concentration (10–30%) and carbonation temperatures around 600 °C.^33,34^ These conditions are substantially different than those used in the CaL-CSP integration, where calcination is conducted at ∼750 °C in pure N_2_ and carbonation is carried out at ∼850 °C in 100% CO_2_. The effect of steam addition during calcination has been previously studied in these latter conditions,^52^ but the role of steam when it is injected during carbonation remains to be revealed. Understanding the influence of steam on both stages is key to finding the operating conditions that may lead to significant improvement of the multicyclic CaL performance.

In the present work, we study the effect of injecting steam during the carbonation stage on the multicyclic performance of limestone when cycled under conditions relevant for CaL-CSP, involving high temperature and 100% CO_2_ in the carbonation stage, and compare the results with those obtained in multicyclic tests conducted with steam under CCS conditions.

## 2 Experimental Section

**Figure 1:**
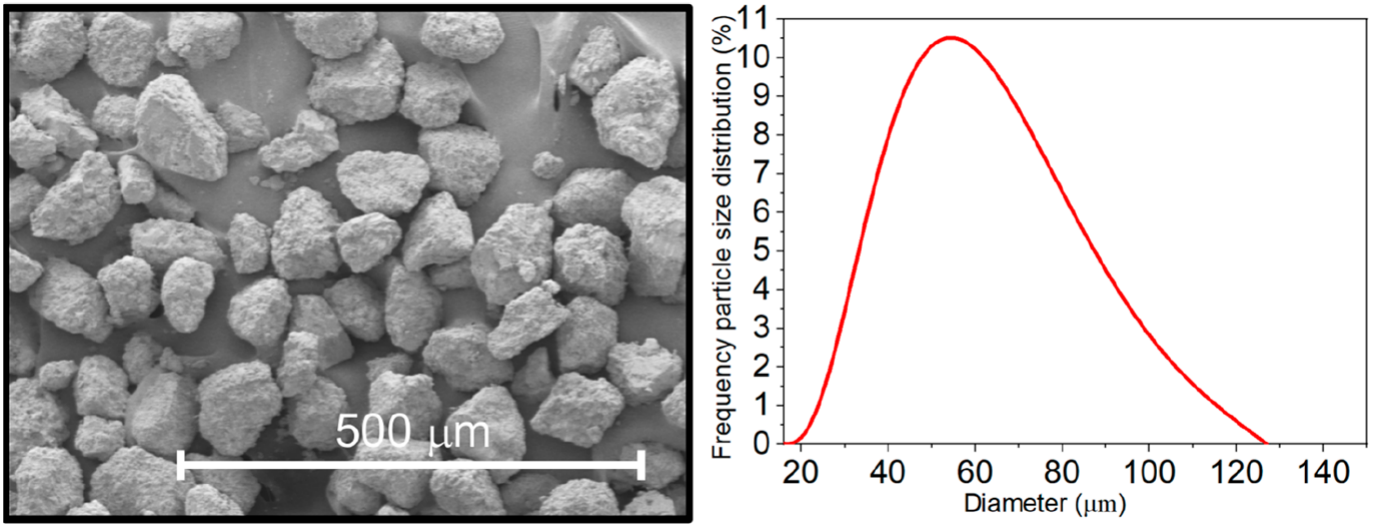
SEM image and particle size distribution (PSD) of the limestone particles tested in the present work.

The limestone tested in this work (ESKAL 60) was provided by KSL Staubtechnik GmbH (Germany) with a particle size distribution (PSD) obtained by aerodynamic classification. Figure 1 shows a SEM micrograph of ESKAL60 and its PSD. The micrograph was taken utilizing a scanning electron microscope HITACHI S4800, while PSD was determined by laser diffractometry as described in ref (63). As may be seen, the sample shows a PSD that peaks around 60 μm. It is well-known that particles’ size can strongly condition calcination and carbonation kinetics, as well as the multicyclic performance.^64^

**Figure 2:**
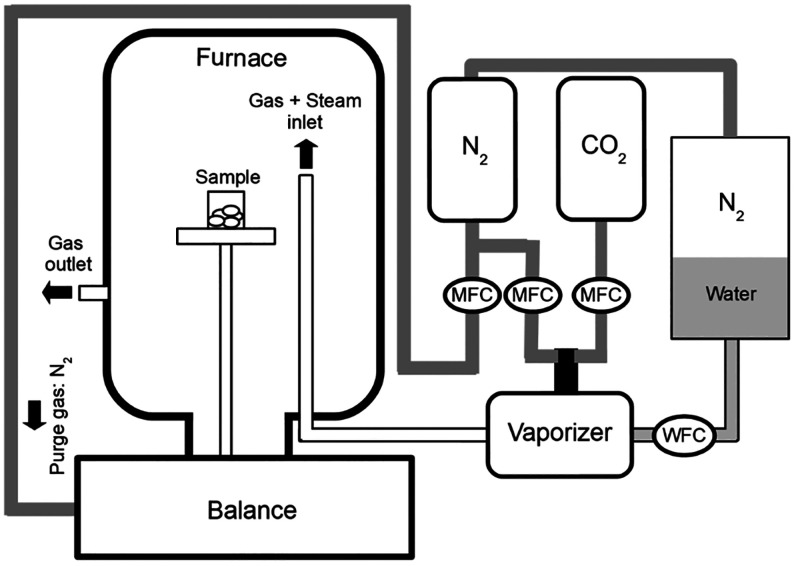
Schematic illustration of the experimental setup.

Figure 2 depicts the experimental setup used. The multicyclic tests were carried out in a thermogravimetric analyzer (TGA) Linseis STA PT 1600 customized for steam injection. To generate steam, water was injected in a vaporizer using a water flow controller (WFC) Bronkhorst Liqui-Flow L13 V12 that allows control of the flow with an accuracy of 1%. Nitrogen was employed to pressurize the water tank and as a purge gas. Steam was mixed with the carrier gas in the vaporizer. The mixture was then injected into the furnace through a heated line kept at 165 °C to avoid condensation. The gas flow rate was controlled using mass flow controllers (MFCs) El-Flow/Bronkhorst. Depending on the target conditions, the carrier gas was N_2_, CO_2_, or a mixture of both.

The CaL-CSP multicyclic tests were devised to imitate reaction conditions relevant in a CaL-CSP operation. Experiments started with a heating ramp of 20 °C/min from room temperature up to the target calcination temperature (730 °C). Calcination lasted 10 min and was carried out in pure N_2_. After calcination, the sample was again increased at a heating rate of 20 °C/min up to the target carbonation temperature: either 800, 830, or 850 °C in different tests. The carbonation reaction was carried out in 5 min long stages conducted under mixtures of either H_2_O/CO_2_ or N_2_/CO_2_. Different values of CO_2_ partial pressure were tested. At the end of the carbonation stage, steam (or N_2_) was removed from the furnace and the temperature was decreased down to 730 °C under CO_2_. At this temperature, CO_2_ was replaced by N_2_ to start a new calcination stage, and the cycle was repeated 20 times.

In the CaL-CCS experiments, calcinations were carried out for 10 min at 900 °C under a 60% CO_2_/40% N_2_ vol/vol atmosphere. Carbonation was carried out at 650 °C for 5 min under 15% CO_2_, using either 0% or 10% steam balanced up to 100% with N_2_. Heating and cooling rates were the same as for CaL-CSP experiments. All the tests were carried out at an absolute pressure of 1 bar.

Scanning electron microscopy (SEM) Hitachi S4800 was used to analyze the impact of sintering on the surface of the particles when the sample was subjected to different conditions. Before SEM, the samples were gold-coated utilizing an Emitech K550 Telstar sputter-coating machine (30 s, 30 mA).

*S*_BET_ surface area and pore size distribution of CaO after one cycle, carried out in different atmospheric compositions, was determined by N_2_ physisorption analysis. In order to minimize measuring errors, a sample of 1 g was cycled in a tubular furnace in conditions that mimicked those used in the multicyclic experiments conducted in the TGA. In these experiments, the sample was first heated at 10 °C/min up to 730 °C, and the temperature was maintained constant for 30 min to carry out calcination in N_2_. Then, the temperature was raised at 10 °C/min up to 850 °C and kept constant for 10 min and the atmosphere changed to conduct the carbonation reaction. Three atmospheres were employed: 29% N_2_/71% CO_2_, 29% steam/71% CO_2_, and 100% CO_2_, with a flow rate of 80 cm^3^. Water was injected into the tubular furnace through a peristaltic pump. Once carbonation was completed, the temperature was decreased down to 730 °C and the sample was calcined again in N_2_ for 30 min. Before the physisorption analysis, the samples were degassed at 350 °C for 2 h.

## 3 Results and Discussion

### 3.1 Influence of Steam Injection during Carbonation on Multicyclic Performance in CaL-CSP Conditions

**Figure 3:**
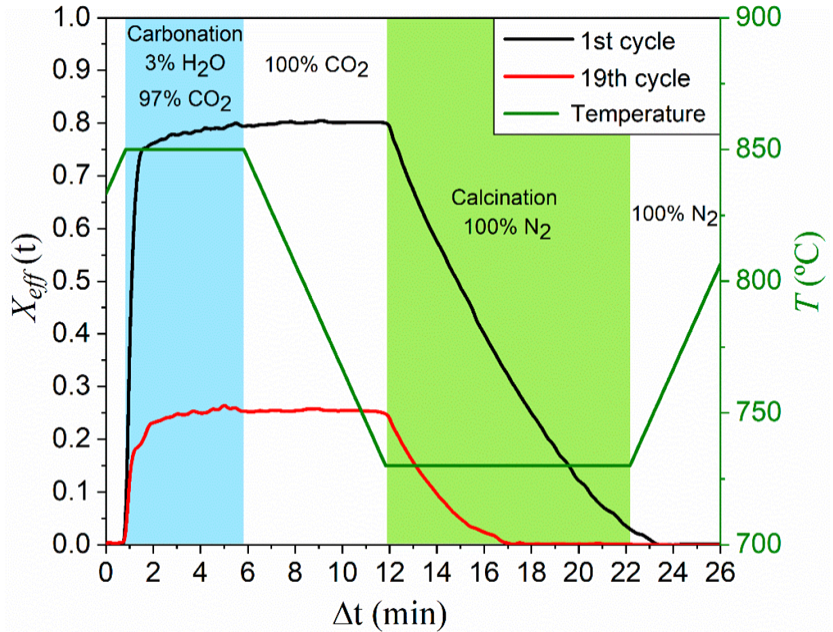
Time evolution of temperature and effective conversion during the 1st and the 19th cycles measured in experiments carried out under CaL-CSP conditions. Carbonation was conducted at 850 °C with a steam partial pressure of 3%.

Figure 3 shows the time evolution of effective conversion *X*_eff_ attained during the 1st and the 19th cycles measured in TGA tests carried out under CaL-CSP conditions. Carbonation was performed at 850 °C with a steam partial pressure of 3%. Qualitatively similar profiles were obtained for the different CO_2_/H_2_O ratios tested. Effective conversion is defined as the quotient between the mass of CaO converted to CaCO_3_ and the total mass of the sample *m*, which includes inert solids if present:
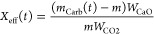
2being *m*_Carb_(*t*) the sample mass at time *t*, and *W*_CaO_ and *W*_CO2_ the molar masses of CaO and CO_2_, respectively.

As expected, effective conversion decreases progressively with the cycle number arguably due to the sintering of the surface plugging carbonate layer.^65^ Two well-differentiated phases can be observed as carbonation proceeds. In the first stage, carbonation occurs rapidly at the CaO particles’ surface. This reaction-controlled phase is followed by a significantly slower phase in which CO_2_ diffuses through the CaCO_3_ layer built upon the CaO surface during the prior stage.^22,66^ Contrarily to what occurs in CaL-CCS conditions, when slow but non-negligible carbonation takes place during this second stage,^36^ at the high temperatures in the CO_2_-rich atmosphere used in CaL-CSP, the formation of the blocking layer impedes any relevant carbonation during the diffusive stage.^20,65^ Thus, as may be seen in Figure 3, most of the reaction occurs during the reaction-controlled fast phase.

**Figure 4:**
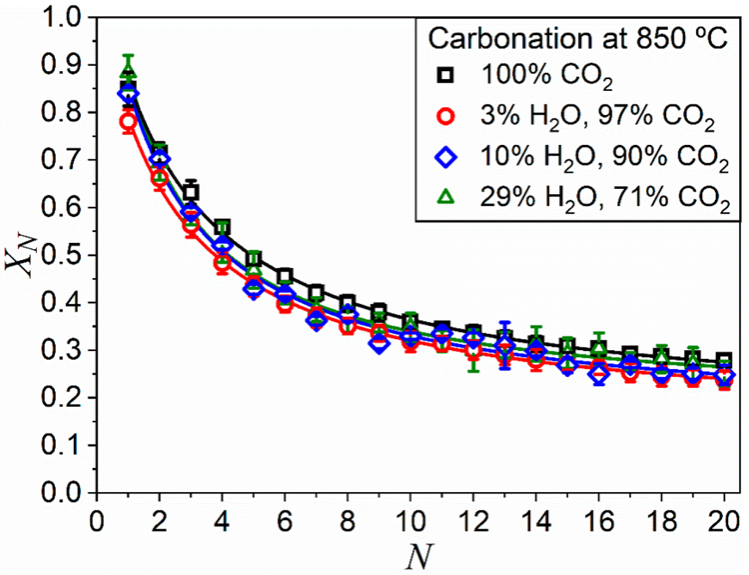
Values of conversion at the end of the carbonation stage as a function of the cycle number obtained from multicyclic experiments at CaL-CSP conditions. Carbonation was carried out at 850 °C in all cases, in atmospheres with different steam/CO_2_ gas mixtures. Lines correspond to the fit of the experimental conversion values to eq 3.

Figure 4 compares data of conversion attained at the end of the carbonation stage as a function of the cycle number for experiments in which the carbonation stage is carried out in different gas mixtures. Multicycle CaO conversion has been calculated using eq 2 and considering the mass converted at the end of the 5 min carbonation stage, which corresponds with the blue area in Figure 3.

Data of multicyclic conversion can be well-fitted by the semiempirical equation:^67,68^
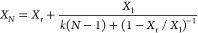
3where *X*_r_ is the residual conversion, *k* the deactivation constant, and *X*_1_ is the conversion at the first cycle. Fitting curves are represented as solid lines in Figure 4. The best-fitting parameters are collected in Table 2.

**Table 2:** Best-Fitting Parameters of Equation 3 to Multicycle CaO Conversion Data (Figures 4 and 6), Corresponding to Multicyclic Experiments Run Using a Carbonation Temperature of 850 °C under Different Gas Mixtures

	H_2_O			N_2_		
carbonation atmosphere^a^	*X*_r_	*k*	*R*^2^	*X*_r_	*k*	*R*^2^
100% CO_2_	0.15 ± 0.01	0.32 ± 0.02	0.998	0.15 ± 0.01	0.32 ± 0.02	0.998
97% CO_2_	0.14 ± 0.01	0.34 ± 0.02	0.998	0.16 ± 0.01	0.33 ± 0.01	0.999
90% CO_2_	0.15 ± 0.01	0.38 ± 0.04	0.998	0.21 ± 0.01	0.36 ± 0.02	0.997
71% CO_2_	0.18 ± 0.01	0.46 ± 0.03	0.997	0.31 ± 0.01	0.33 ± 0.01	0.999

a The carbonation atmosphere is balanced up to 100% with either H_2_O or N_2_ as indicated in the first row.

Since the material is expected to be cycled many times, the most relevant parameter for practical purposes is the residual conversion. No significant influence of steam amount is observed in these experiments on the residual conversion values, which are similar regardless of the steam/CO_2_ ratio employed. Nevertheless, the deactivation constant increases with the amount of steam, which might indicate that steam promotes the rate of sintering.^16,69^

**Figure 5:**
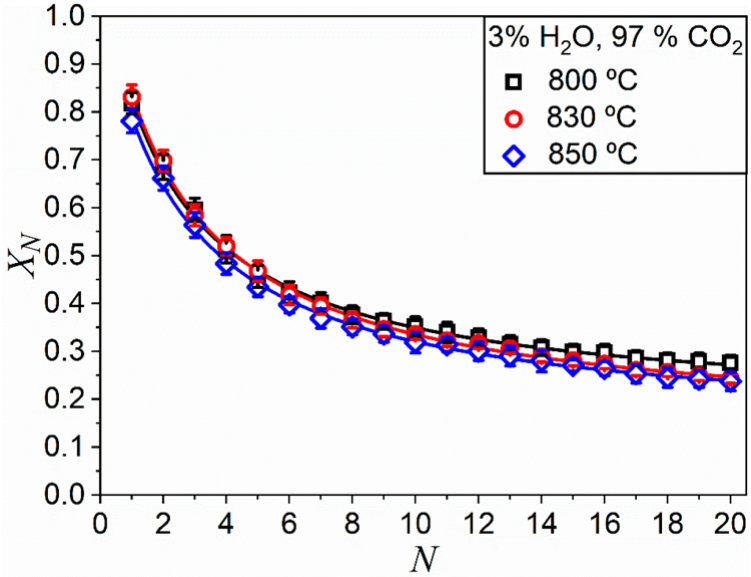
Multicyclic CaO conversion obtained from tests carried out by carbonation at different temperatures under a 3% H_2_O/97% CO_2_ atmosphere.

Figure 5 illustrates the influence of the carbonation temperature in the presence of steam. It includes data on the CaO multicyclic conversion obtained from experiments carried out at diverse carbonation temperatures for a fixed atmosphere composition (3% H_2_O/97% CO_2_). The best-fitting parameters of eq 3 to these data are shown in Table 3. While modest, a slight improvement of the multicyclic conversion is observed when carbonation temperature is reduced.

**Table 3:** Best-Fitting Parameters of Equation 3 to the Experimental Data Presented in Figure 5, Corresponding to Multicycle Experiments Run Using Different Carbonation Temperatures but a Fixed Atmosphere Composition (3% H_2_O/97% CO_2_)

temperature (°C)	*X*_r_	*k*	*R*^2^
800	0.18 ± 0.01	0.39 ± 0.02	0.998
830	0.14 ± 0.01	0.33 ± 0.01	0.999
850	0.14 ± 0.01	0.34 ± 0.02	0.998

**Figure 6:**
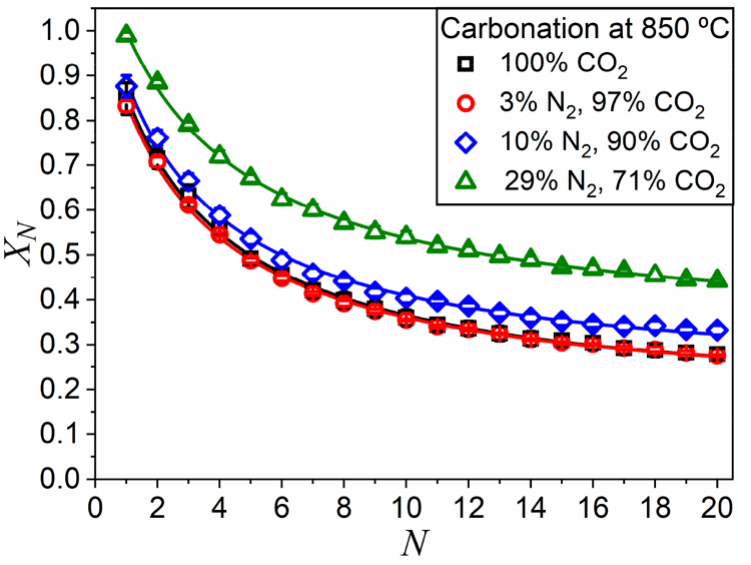
Multicyclic conversion obtained from tests in which carbonation was carried out at 850 °C, in atmospheres that contained different CO_2_/N_2_ gas mixtures as indicated. Solid lines correspond to the best fit of eq 3 to the experimental data.

It should be taken into account that the injection of steam has a dilution effect, reducing the percent CO_2_ in the atmosphere, thereby modifying the thermodynamic equilibrium temperature. Since carbonation is carried out at high temperatures close to equilibrium a slight change of the CO_2_ volume percent may have a relevant influence on both the reaction kinetics and on the morphology of the arising CaCO_3_ particles.^70^ Therefore, to better assess the role of steam during carbonation, multicyclic tests were carried out using N_2_/CO_2_ gas mixtures instead, keeping the same CO_2_ concentrations used in the previous H_2_O/CO_2_ gas mixtures. Experimental results are depicted in Figure 6. The values of residual conversion and deactivation constant corresponding to these experiments are collected in Table 3. In contrast to what was observed with steam, the dilution of CO_2_ with N_2_ noticeably improves the multicyclic performance of the sample. Residual conversion values derived from the experiments under N_2_/CO_2_ are consistently higher than values observed in the corresponding H_2_O/CO_2_ mixtures.

**Figure 7:**
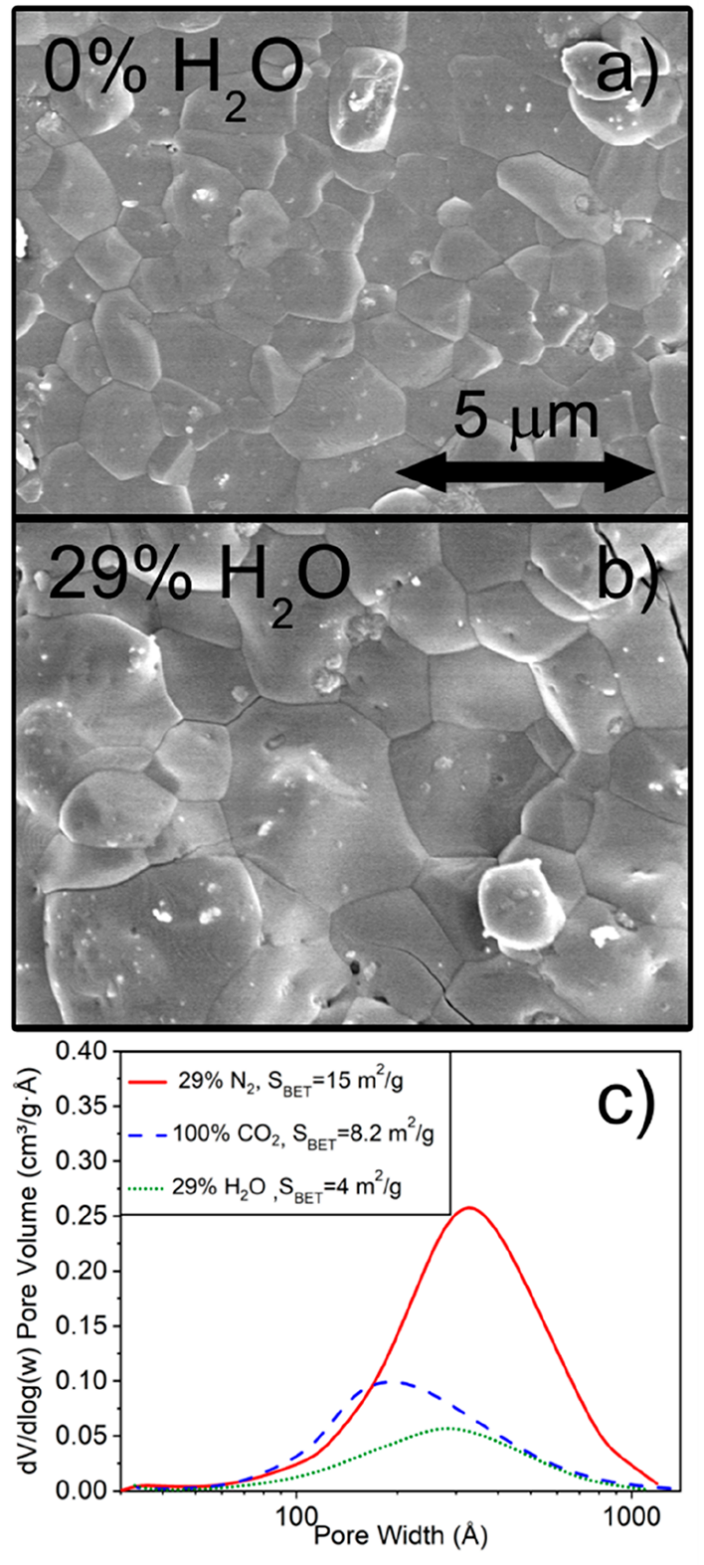
SEM micrograph taken after the first carbonation conducted with a) 0% H_2_O and b) 29% H_2_O. c) Pore size distribution and BET surface measurements of CaO after one cycle carbonating in different atmosphere compositions: 100% CO_2_, 29% H_2_O/71% CO_2_, and 29% N_2_/71% CO_2_.

Data plotted in Figure 6 indicate that diluting CO_2_ with N_2_ has a positive impact on the multicyclic performance. The reduction in the proportion of CO_2_ in the sample environment alleviates the sintering-induced deactivation. However, when dilution is done in steam no positive influence on the multicycle activity is observed. This could be explained by the mineralizing effect of steam, which at such high temperatures accelerates the loss of reactivity, and adversely compensates for the positive effect of the dilution. The impact of H_2_O on CaCO_3_ is observed in Figure 7, which shows two SEM micrographs taken after the first carbonation, conducted with no steam (Figure 7.a) and with 29% H_2_O (Figure 7b). With 29% H_2_O, the CaCO_3_ grains on the particle’s surface are noticeably larger.

Figure 7c shows a porosimetry analysis of the nascent CaO after carbonation and calcination cycle, carried out in different atmospheres: 100% CO_2_, 29% H_2_O/71% CO_2_, and 29% N_2_/71% CO_2_. The results of BET surface measurements are included in the legend. Given that the microstructure of the nascent CaO essentially depends on that of the original CaCO_3,_ the porous structure of the nascent CaO provides information about the degree of sintering attained in the previous carbonation stage.^28,64^ As expected, the measurements prove substantial sintering after carbonation is carried out in the mixture H_2_O/CO_2_, while the loss of reactive area and porosity is less pronounced when the reaction is conducted in N_2_/CO_2_. These results are consistent with the values of conversion attained in the second cycle and the SEM micrographs in Figure 7.

**Figure 8:**
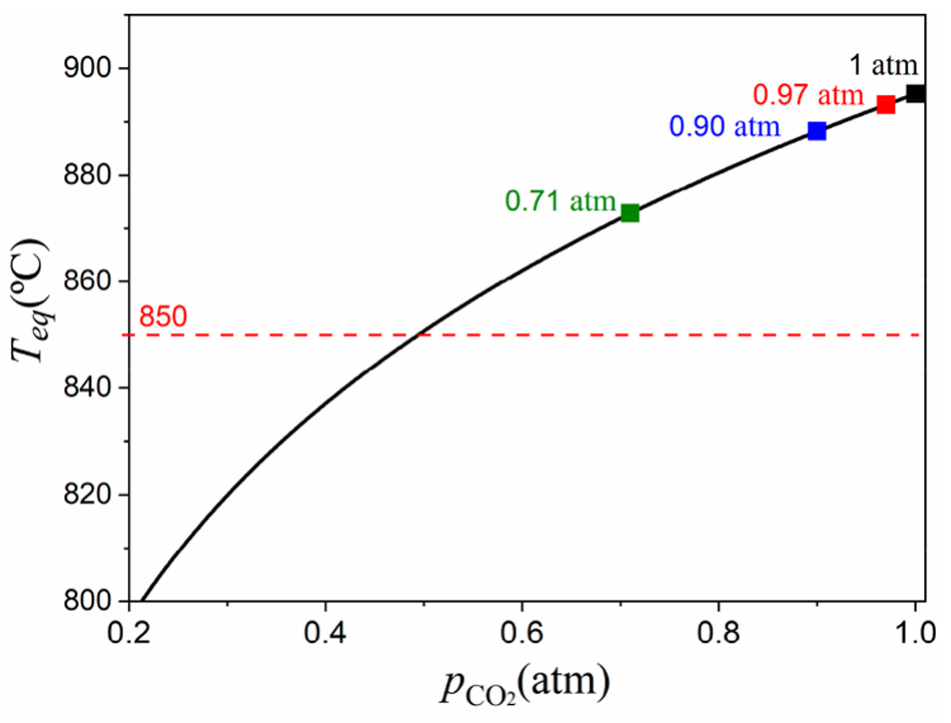
Equilibrium temperature as a function of the CO_2_ partial pressure. Indicated as colored points are the points corresponding to the values of partial pressures of CO_2_ used in our experiments.

**Figure 9:**
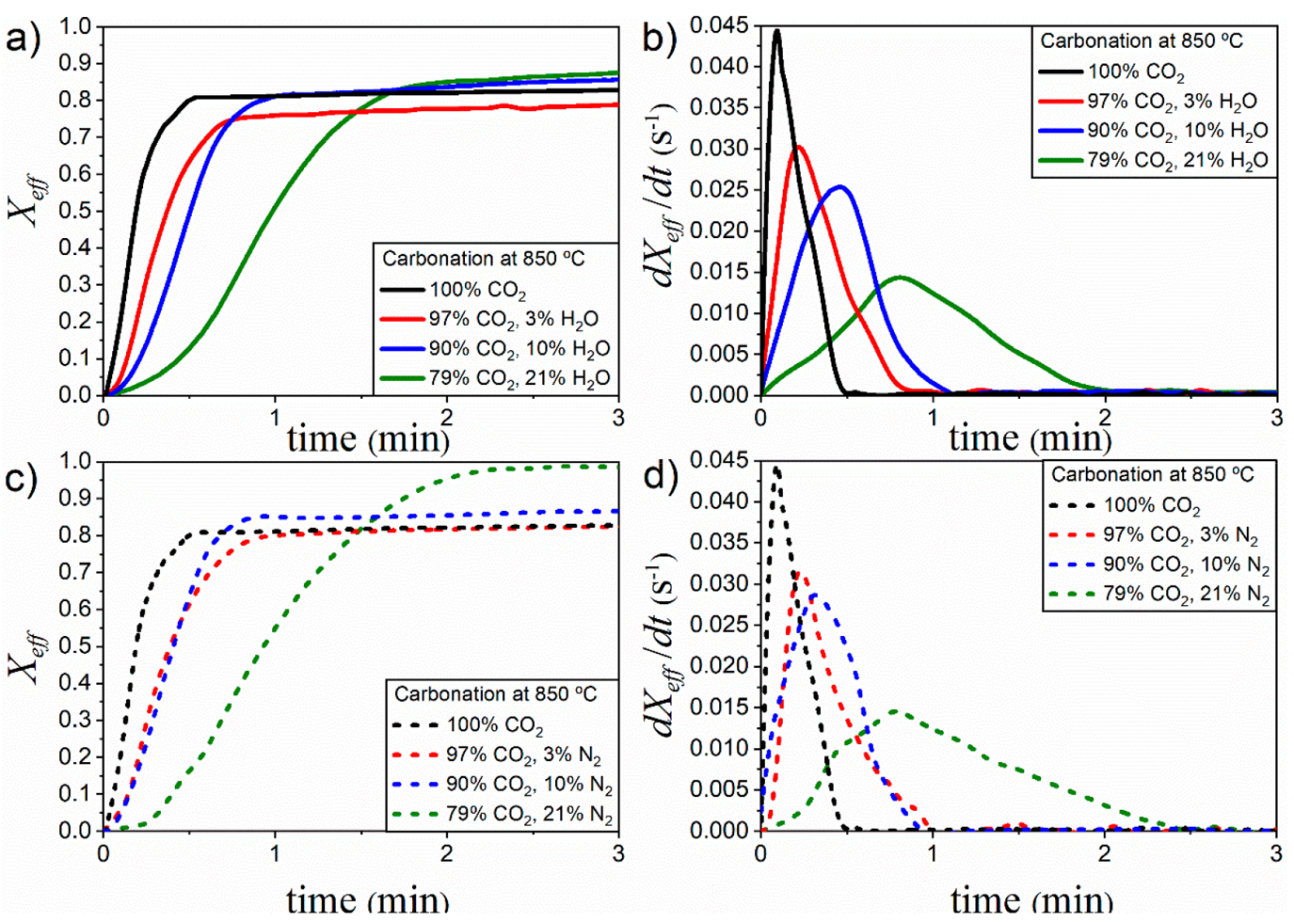
Time evolution of effective conversion and its derivative during carbonation in atmospheres of different gas mixtures for the first cycle.

An additional aspect of the CO_2_ dilution that ought to be considered is the change of the equilibrium temperature of the reaction, which decreases with the CO_2_ concentration. According to thermochemical data, the relation between the equilibrium temperature and the CO_2_ partial pressure is^69−71^
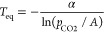
4where *A* = 4.083 × 10^7^ atm, α = 20 474 K, and *p*_CO2_ is given in atm. This equation is plotted in Figure 8. As the CO_2_ partial pressure is decreased by the addition of steam or N_2_, the equilibrium temperature approaches the target carbonation temperature used in the multicyclic tests under CaL-CSP conditions (*T* = 850 °C), resulting in slower carbonation kinetics, as proven in Figure 9.

Figure 9 shows the time evolution of effective conversion during the first carbonation at 850 °C as well as its derivative, d*X*_eff_/d*t*, under different gas mixtures. As expected, the rate of carbonation decreases with decreasing CO_2_ partial pressure. The maximum reaction rate is approximately the same regardless of the used gas to dilute the CO_2_. Thus, carbonation kinetics during the fast kinetic-controlled stage is not influenced by steam but depends exclusively on the partial pressure of CO_2_.

### 3.2 Influence of Steam Injection during Carbonation on Multicyclic Performance in CaL-CCS Conditions

At first sight, the results presented in the previous section contrast with several previous studies reporting that the use of steam during either carbonation or calcination (or in both) enhances the CaL multicyclic activity of limestone derived CaO.^33,34,36,53,58^ However, previous studies were all performed under reaction conditions compatible with CaL-CCS, involving carbonation under relatively low temperature and low CO_2_ concentration, while the tests presented above involve carbonation at high temperature and high CO_2_ concentration. Under these conditions, the results depicted in Figure 4 show that steam injection during carbonation does not improve the multicyclic performance. Indeed, the effect of steam appears to be even detrimental if we compare the values of residual conversion when CO_2_ is diluted with steam with those in which CO_2_ is diluted with N_2_ at the same CO_2_ concentration (Table 2). This could be explained by the high carbonation temperatures and high CO_2_ concentration values employed for carbonation in CaL-CSP as compared to the conditions used in CaL-CCS. Under such harsh conditions, particle sintering is substantially promoted. Moreover, as a mineralizer agent, the presence of H_2_O further promotes grain growth and particle sintering.^16^ The adverse effect of H_2_O during carbonation at high temperature is also upheld by the results shown in Figure 5 and data collected in Table 3, indicating that the residual conversion decreases with the carbonation temperature.

**Figure 10:**
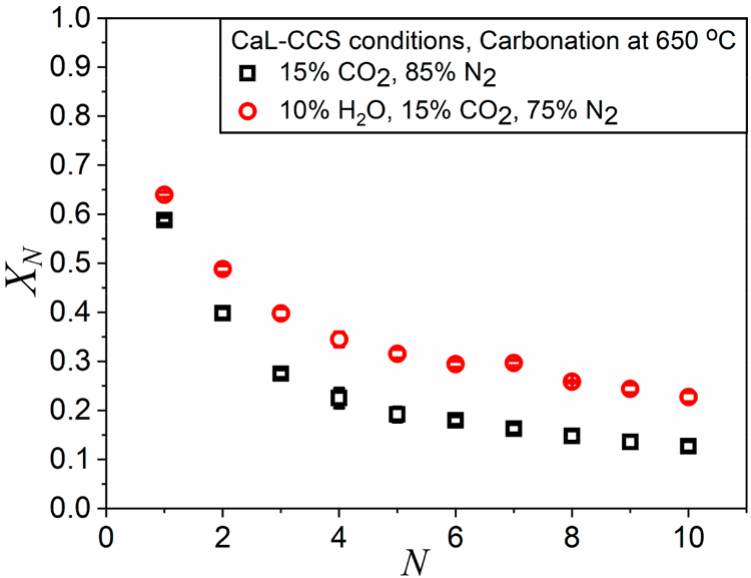
Multicyclic conversion data obtained from tests conducted under CaL-CCS conditions with steam addition during carbonation (red open circles) and with no steam (black open squares).

To further investigate this apparent contradiction, multicyclic tests under CaL-CCS conditions, in which steam was added in the carbonation stage, were also conducted to check whether steam enhances in these conditions the multicycle performance as reported in the literature thereby reinforcing our results. In these experiments, we replicated the experimental conditions employed by Donat et al.^33^ The results here obtained, shown in Figure 10, are very similar to those reported by Donat et al, even though their experimental setup differ from ours; they utilized a bubbling fluidized bed (BFB) reactor instead of a TGA apparatus. The fact that the improvement typically reported in CCS conditions was replicated in our multicyclic tests confirms that steam’s addition during carbonation is indeed beneficial for CaL-CCS but neutral under CaL-CSP conditions. This contrast may be ascribed to the different carbonation conditions regarding temperature and CO_2_ partial pressure.

**Figure 11:**
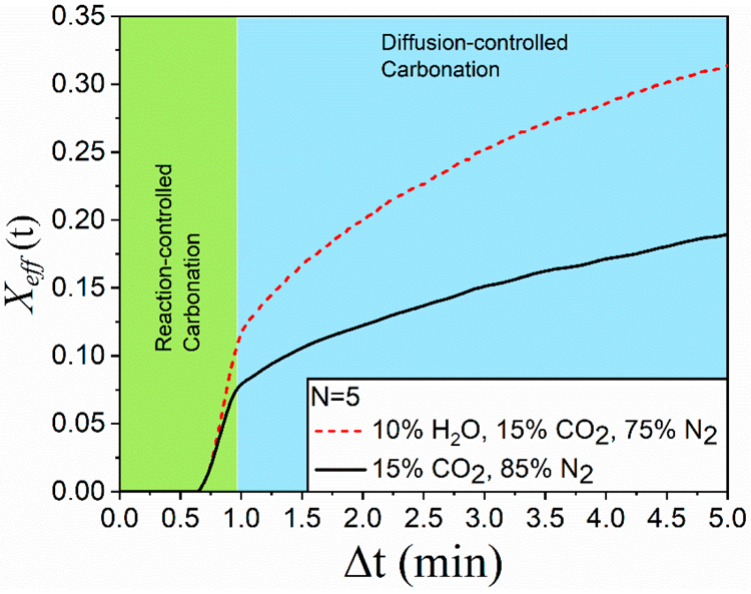
Time evolution of effective conversion during carbonation at the fifth cycle from tests carried out in CaL-CCS conditions.

Time evolution of conversion during the fifth cycle, obtained in our work from the experiments conducted in CaL-CCS conditions, is depicted in Figure 11. Albeit, during carbonation, the positive influence is observed on both the reaction-controlled and the diffusion-controlled phases, the improvement is more pronounced in the latter as it has been previously reported by Manovic et al.^36^ Arguably, the improvement can be attributed to an enhancement in the diffusion of CO_2_ through the CaCO_3_ blocking layer formed during the reaction-controlled stage, favored by the steam. Conversely, as shown in Figure 3, conversion during the diffusion-controlled phase is negligible in TCES-CSP conditions, even under steam.

## 4 Conclusion

The results reported in this work show that the effect of steam injection during carbonation on the multicycle activity of limestone-derived CaO strongly depends on the conditions used for carbonation. In agreement with previous results reported in the literature, we found that steam enhances the CaO multicycle activity under carbonation conditions suitable for CO_2_ capture from postcombustion gases (CaL-CCS); moderate temperatures ∼600 °C under an atmosphere with low CO_2_ concentration. Under such conditions, steam significantly enhances the conversion during the diffusion-controlled stage, which accounts for a large share of the total conversion attained. Arguably, the presence of steam favors solid-state diffusion of the CO_2_ across the CaCO_3_ layer built upon the CaO particles in the reaction-controlled phase of carbonation. Conversely, when the carbonation reaction is carried out at high temperatures (over 800 °C) in a CO_2_-rich atmosphere, overall, the addition of steam does not alter the multicyclic performance. The slight benefit gained by the effective dilution of CO_2_ by the addition of steam is offset by the mineralizing effect of steam, which noticeably promotes particle sintering, harming CaO reactivity and probably the diffusion of CO_2_ toward the inner unreacted core of the particles. The influence of steam on carbonation kinetics is also different at temperatures over 800 °C in CO_2_-rich atmospheres. Under such conditions, the fraction of CaO converted during the diffusion-controlled phase becomes negligible and it is not improved by steam. Finally, the results herein show that that carbonation kinetics are essentially governed by the partial pressure of CO_2_, as similar reaction rates are observed regardless of whether the CO_2_ is diluted in steam or N_2_.

However, the CaL-CSP integration for thermochemical storage of solar energy benefits from the rapid and extensive carbonation attained at high temperatures in CO_2_ Therefore, since steam is beneficial when injected during calcination but not during carbonation, any application of steam to CaL-CSP should be constrained to the calcination stage.
